# Food and Nutrition-Related Knowledge, Attitudes, and Practices among Reproductive-age Women in Marginalized Areas in Sri Lanka

**DOI:** 10.3390/ijerph17113985

**Published:** 2020-06-04

**Authors:** Permani C. Weerasekara, Chandana R. Withanachchi, G. A. S. Ginigaddara, Angelika Ploeger

**Affiliations:** 1 Specialized Partnerships in Sustainable Food Systems and Food Sovereignty, Faculty of Organic Agricultural Sciences, University of Kassel, 37213 Witzenhausen, Germany; a.ploeger@t-online.de; 2 Department of Archaeology and Heritage Management, Faculty of Social Sciences and Humanities, Rajarata University of Sri Lanka, Anuradhapura 50000, Sri Lanka; chandanawithanachchi@gmail.com; 3 Department of Agricultural Systems, Faculty of Agriculture, Rajarata University of Sri Lanka, Anuradhapura 50000, Sri Lanka; sanjeewanieg@gmail.com

**Keywords:** food-and nutrition-related knowledge, attitudes and practices, reproductive-age women, marginalized society, nutrition and health problems, food and nutrition security, Sri Lanka

## Abstract

Nutrient deficiencies are a public health problem in Sri Lanka. Achieving food security is a major challenge due to unhealthy eating patterns. The nutritional status of a woman and her knowledge is a powerful indicator of the nutritional security of her children and household food security. Nutrition-related knowledge and attitude are necessary for dietary changes towards a healthier dietary pattern. For that reason, food and nutrition-related Knowledge, Attitude and Practice (KAP) is one of the key factors to achieving household food and nutritional security. The main objective of this study is to assess the food and nutrition-related KAP among reproductive-age women and understanding of household food and nutritional security in Sri Lanka as an example for marginalized societies. Thus, a cross-sectional survey was conducted using the KAP model questionnaire administered on 400 reproductive age women (18–49 Years) in marginalized areas in Sri Lanka. Data were collected using a random sampling method. The research results clearly showed that the reproductive age women have a low level of nutritional knowledge in the areas being investigated. Most women have a positive attitude towards receiving nutritional knowledge but have low-level practice about a healthy diet. Furthermore, knowledge, practices, and attitudes of women largely affect their BMI status, as well as household food security. Multiple linear regression analysis was used to analyze the influential factors. There was a highly significant positive correlation between nutritional knowledge, attitude score, and BMI level and a significant difference was found in the area, age, family size, monthly income, educational level, attitudes towards nutrition, food and nutrition practices across the reproductive women (R^2^: 467, *p* < 0.01). The research results showed that KAP largely determines women’s nutrition and household food security. Based on the results of this research, there is a need to enhance nutritional education in reproductive-age women in marginalized areas in Sri Lanka.

## 1. Introduction

Food insecurity is a global problem that contributes to poor health and nutritional deficiencies. It can affect health either directly or indirectly through nutritional status as indicated by undernutrition or overnutrition [1] and it is related to macro- and micronutrient deficiencies and lack of dietary diversity [1,2]. Also, women’s nutritional status has been identified as an indicator of the overall well-being of society [3,4,5] and the nutritional security of children [6,7]. Hence, Reproductive-age women’s nutritional status is the single most important criterion influencing pregnancy outcomes [8]. Inadequate and improper dietary-intake patterns in women of reproductive age result in the deficiency of essential nutrients [9]. According to the World Health Organization (WHO), many women do not get enough micronutrients in their diets during their reproductive-age period [10], which influences future generations. Unhealthy eating habits [11,12], such as consuming nutrient-deficient food [13,14], skipping meals [15,16], and a lack of proper eating patterns [17], are understood to cause various health problems and nutritional deficiencies [18]. Therefore, knowledge about healthy food choices is a factor for maintaining a healthy diet [13,19].

In Sri Lanka, 17% of newborn babies have a low birth weight, and one-sixth of women have a low body mass index [20]. The prevalence of anaemia is 16.2% among pregnant women and 19.6% among lactating women [21,22] 32.6% of women of reproductive age have anaemia [23] Vitamin A deficiency is observed in approximately 15 % of mothers with children aged 6–60 months [24]. Other micronutrient deficiencies such as iron, iodine, zinc and vitamin D are reported in different age groups [24,25,26]. According to WHO 2018 for Sri Lanka, Non-Communicable Diseases (NCDs) such as cancer, chronic respiratory diseases, cardiovascular diseases and diabetes, are estimated to account for 83% of all deaths [27,28]. This nutrition statistics indicate problems associated with low-quality food intake and unhealthy eating patterns in Sri Lanka. If women are aware of healthy nutrition, they can help minimize the occurrence of many nutritional problems. Hence, women’s education is associated with positive effects on family nutritional status [29,30,31]. Previous studies suggested that women’s nutritional knowledge of traditional food could also impact the nutritional status of the family [14,32,33]. Improving women´s food- and nutrition-related knowledge has a positive influence on health [34,35]. Women´s low level of nutritional knowledge impact on nutritional problems [36] and a healthy diet and nutrition education programs help to improved health outcomes among reproductive women [37]. Also, studies suggested that a proper nutrition knowledge impact on good nutrition status [38]. Insufficient nutrition-related knowledge of women is an underlying cause for the high prevalence of undernutrition and micronutrient deficiencies [39,40,41], as women’s nutritional knowledge affects attitudes and eating behaviour [14]. One of the studies suggested that women’s inadequate nutrition knowledge and their food intake did not meet all the nutritional requirements of pregnancy [42]. Sufficient women’s diet-related knowledge is needed for an individual to evaluate the quality of their own and their family’s diet [43]. A study indicated that women with higher education levels have the highest level of nutritional knowledge related to folic acid and iodine deficiencies during pregnancy [32,44]. Likewise, the role that women play in terms of household food security is not very different from that in many other developing countries compared to the cultural context of Sri Lanka, where women are predominantly assigned the role of food preparation and food management within the household [45]. Women have played a key role in food preparation in Sri Lankan culture since ancient times. Studies suggest that in addition to knowledge for maintaining good health practices [46], nutritional status is affected by positive attitudes and practices [47,48,49]. Therefore, in Sri Lanka, women’s lack of knowledge impacts dietary practice [50] and women’s nutritional knowledge impact on healthy lifestyle [51]. Thus, women’s nutrition knowledge is a key factor in maintaining health and nutrition [51].

In such a context, women’s nutritional knowledge, behavioral attitudes and practices are essential because women mostly control and oversee their entire family’s food consumption in Sri Lanka. Therefore, women’s food and nutrition-related knowledge are important factors to enhance household food security as well as nutritional status. In this situation, achieving an effort to improve nutrition and measuring their impact requires more useful indicators and tools for [52], especially vulnerable societies and food and nutrition-related KAP must be better understood. Hence, KAP surveys reveal misconceptions or misunderstandings that may impede behaviour and conduct barriers to behaviour change [52]. Sri Lanka is in a phase transforming their traditional food systems toward the western diet as well as an unhealthy eating pattern. The best tool to avoid this situation is to spread attitudes through food and nutrition knowledge [53]. Thus, it is important to the understanding of food and nutritional knowledge of women in marginalized areas in Sri Lanka. However, a systematic study of the reproductive age women‘s food and nutrition-related knowledge, attitude practice and their perception of food security and food quality has not been conducted in marginalized societies in Sri Lanka yet. Thus, studies that evaluate and analyse women’s food and nutrition -related KAP are a useful method for gaining such an insight into women’s determinants of their dietary habits. Thereby, this research will help policymakers or responsible authorities to plan appropriate nutrition care programs [52] for vulnerable people living in marginalized areas. These results could be empowering sustainable food and nutrition system they can provide valuable inputs for effective programs and projects. This study examines the following questions: (a). How knowledge attitude and practice among women and is its impact on the nutrition and health situation? (b). What factors influence women’s food and nutrition knowledge? (c). What is the women’s perception of food quality and perceived risk in these areas? Therefore, the main objective of this study is to identify food and nutrition-related KAP among reproductive-age women and to understand the impacts of KAP on household food and nutritional security in marginalized societies in Sri Lanka. For that reason, a cross-sectional survey was conducted using the KAP model questionnaire administered in marginalized areas in Sri Lanka.

## 2. Materials and Methods

### 2.1. Study Area

**Figure 1:**
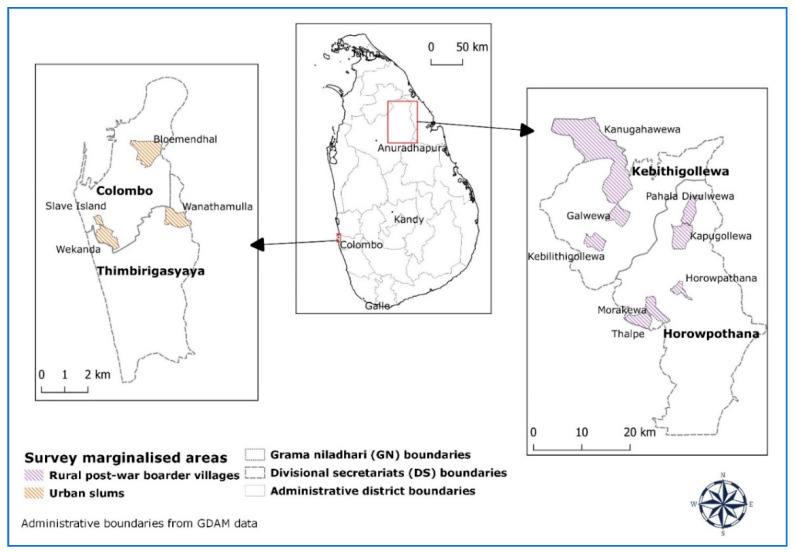
Study map (authors’ illustration).

The research study was conducted in 2017–2019 in two different marginal areas based on the cross-case-characteristics method [54]. This research is part of a collaborative project investigating nutritional transition and traditional food cultural changes in Sri Lanka during colonization and post-colonization [53]. The study was conducted in a remote rural area (Anuradhapura district), and urban slum and shanty areas (Colombo district) due to the entire population being below or near the poverty line. These two different groups were selected based on a high vulnerable status due to low income, and nutritional, social, and health-related problems in different ways. In the north-central province, the district of Anuradhapura was selected as a remote rural study site. The main income for people comes from agriculture, and the main crops are paddy [55]. In Anuradhapura, two divisional sectaries (DS) divisions (Kebithigollewa and Horowpothana) were selected for the present study (see Figure 1).

All divisions’ residents engage largely in small-scale agriculture as their main source of income. In these rural remote areas people suffer from the consequences of 30 years of war (war-border areas) as well as poverty and malnutrition, water problems (dry-zone areas), and kidney diseases. In the western province, the district of Colombo was selected for the urban areas. In Colombo, three GN divisions were selected for this study (see Figure 1). Low-income residents who live in these areas suffer from malnutrition, social and health problems, poverty, inadequate living space, unsanitary conditions, and high rates of unemployment (55).

### 2.2. Study Design and Data Collection

Ethical approval was obtained from the Ethical Review Committee (ERC) of the Faculty of Applied Science of Rajarata University of Sri Lanka (ref: ERC/09/19) for this research project. A cross-sectional survey was conducted using the KAP model questionnaire administered on 400 reproductive age women (18–49 Years) in marginalized areas in Sri Lanka. The study was conducted in December 2018–February 2019. The self-designed questionnaire used in this study was based on the KAP model [52]. Well-trained field workers collected data. Enumerators were trained on how to conduct an interview using questionnaires. Anthropometric measurements were taken for all women in both areas (*N* = 400). All interviews were conducted in the official language (Sinhala) in Sri Lanka. The sample was selected based on gender and age. According to a feasibility study, women are a powerful indicator of household food security in the area, since they are main income holders, but also suffer from nutritional problems. Selected sample size is 400 as it implies in a confidence interval of +− 2.5% (total = 5%). Within both areas, 400 reproductive age women (18–49 years old) were randomly selected from the electoral divisional secretariats (DS) list based on women’s age (18–49 years), comprising 200 rural household´s and 200 urban household´s reproductive-age women. One woman per household was selected. In case a household’s selected candidate refused to participate or no women were available for an interview, then the nearest house’s women were interviewed. All women participated in the survey (*N* = 400, 100%). Incorrect data did not find because questionnaires were filled by the enumerators through face-to-face interviews. No monetary incentive was the provider for the participant. Furthermore, current research tried to understand food and nutrition-related behaviours, perception of food, and women’s perception of food quality and perceived risk. For these purposes, randomly selected women of reproductive age (*N* = 40) were interviewed in the same sample (*N* = 400) from both areas. The interviews were conducted in Sinhala and then translated into English. Interview data were transferred to an Excel datasheet. MAXQDA 2018 was used for coding the interviews. Interview data were also transferred to the datasheet before conducting quantitative statistical analysis. The interview questions were slightly altered based on research location and position. However, it remained connected to the research objectives.

### 2.3. Questionnaire

The self-designed questionnaire was used based on the KAP model and Sri Lankan dietary guidelines. The questionnaire was divided into the following sections:

(1). Demographic Characteristics

Demographic characteristics were based on socioeconomics factors such as type of residence(rural/urban), age (18–49 years old), marital status (married/single/separated/widowed), family size base on the household members (small family/big family), main occupation (unemployed/agriculture/hard job/government job), monthly family income, food expenditure and level of education (non-education/primary/secondary/higher education), residence period (from birth/immigrated from somewhere).

(2). Anthropometric Measurements

Anthropometric measurements were taken using a stadiometer and electronic weighing scale. Height was measured to the nearest centimetre by using a height scale following the standard anthropometric technique for weight and height measurements. Participants were asked to remove their shoes and wear light clothing [56]. Body mass index (BMI) was calculated by dividing weight (kilograms) with height in meters squared (kg/m^2^). Evaluation of nutrition status (undernutrition, overnutrition, and obesity) using BMI was based on the following WHO criteria [57]: BMI < 18.5, underweight (malnourished); BMI = 18.5–24.99, normal weight; BMI = 25–29.99, overweight; and BMI > 30, obesity [58]. The calculation of BMI will help better to understand the nutrition situation of the research areas

(3). Food and Nutrition-related Knowledge, Attitude and Practice (KAP)

Food and nutrition-related KAP data were collected using a questionnaire according to FAO guidelines [52,59,60]. A nutrition-related-knowledge questionnaire that contained seven parts and 50 questions and nutrition-related attitude questionnaire that contained five-part and 20 questions were used to comprehensively assess the level of women’s food and nutritional knowledge (see Table 1).

The score of nutrition-related knowledge and attitude were calculated using preliminary analysis. Each question has been judged as yes or no. The women in the study were asked to assess their attitudes towards good diet practices and if they were concerned about what they were eating. Eating habits like eating in front of the TV, eating with other family members, eating breakfast, and dietary practices were also used to assess the influence of the home environment on diet practices. Women were asked if they were concerned about what they were eating to assess attitudes towards good diet practices and the aforementioned eating habits like eating in front of the TV and with other family members. Dietary-practice data were collected using a questionnaire to include the number of meals consumed daily, meal patterns, snacking habits, food flavours, food quality.

(4). Knowledge about Food Quality and Perceived Risk

These questions were based on women’s knowledge of food quality and perceived risks, especially since both areas are facing the problem of food quality. Basic knowledge about food quality such as what is recommended as healthy or unhealthy food by governmental associations are not known.

### 2.4. Data Analyses

Data were analysed using SPSS Statistics Version 21.0 (IBM, Armonk, NY, USA). Invalid or missing data were excluded and all data entries were double-checked to prevent errors. Descriptive statistics, such as frequency and percentage were used to analyse demographic factors. *t*-tests, ANOVA, Mann Whitney were used to compare differences in variables. Multiple-linear-regression models were established to evaluate factors that influenced nutrition-related knowledge. VIF is used for diagnosing multicollinearity or collinearity. High values signify that it is difficult to impossible to assess accurately the contribution of predictors to a model. A VIF < 5 implies no multicollinearity [73]. All statistics were checked using a two-sided test. Statistical significance was fixed at *p* < 0.05. A significant relationship between nutrition-related KAP and BMI status was determined by using the chi-squared test, correlation.

## 3. Results

### 3.1. Demographic Characteristics of Reproductive-Age Women

In the study sample, most women were 15–35 years old (70.6%). Most women were married (82%), and 91.3% of the participants were educated, of which most had at least primary education (58.5%), while 60.5% were housewives without incomes. According to Sri Lankan socio-economic data, median female age was 34.4 years [74,75] while female literacy rate was 91% [76,77]. The average monthly household median income was between 15,000–18,313 LKR. [78,79] which is compared with this sample found a low percentage and It was 25% (15,001–25,000 LKR). However, according to the study sample, many of the monthly household income was between 5000–25,000 LKR. Regarding the nutritional status of reproductive age women, 43.5% were underweight, and 74% of the interviewed women have lived in their respective villages since birth or for at least a decade. The study found that, in urban areas, about 50% of the participants were underweight, 20% overweight, and 5.5% obese. On the other hand, 42.5% of the examined women in rural areas had normal weight compared with 24.5% of those in urban areas. The study found that fewer than 50% of the women had normal weight, and more than 50% of the women had nutritional problems such as under- and overnutrition. The nutritional status of rural women was significantly better than that of urban women (see Table 2).

### 3.2. Food and Nutrition-Related Knowledge

The average score of food and nutrition-related knowledge of reproductive women based on age, area, household size, marital status, main occupation, monthly income, level of education and BMI. The mean score of nutrition-related knowledge was 0.40 ± 0.507. 

The lowest average score was “Nutrient and food-related diseases” and the highest average score was “Dietary fibre related knowledge”. The statistically significant difference was found between age, household size, area, monthly income, main occupation, marital status, education and BMI (*p* < 0.05). The average score of participants aged more than 25 years old was highest in each part. The average scores of married women were higher than the other participants. The average scores of normal-weight participants were lower than others categorised as underweight, overweight and obese. The average scores of those with education were higher than the non-education score. The average scores of employed women were higher than unemployed women.

The mean score of participant women aged more than 35 years old was high, whereas the mean average score of participant women aged 18–25 years old was lower. The average score of the big family participants was higher than for small family participants. The average score of knowledge for women living in rural areas was higher than those living in urban areas. The average score of hard job participants was higher than those of government job participants. The average score of the higher education knowledge level was higher than those with secondary and primary education. The average score of underweight women was the highest, and the score of women who were overweight was the lowest Meanwhile, the average score of women who have normal weight and related micronutrients knowledge, nutrient and food-related diseases and healthy food-related knowledge were higher than women who had to suffer from undernutrition and overnutrition as well as obesity (see Table 3).

Of the 400 women, the majority (71.75%) did not know about vitamins and minerals. Most of the women (80.5%) responded incorrectly to food and nutrition diseases, and the majority (57.2%) did not know what a balanced diet was. Three-quarters of the women (75%) did not know that diets low in iron cause anaemia, while 94.6% did not know which minerals and vitamins were good for bones and brain development (see Table 4).

### 3.3. Food and Nutrition-Related Attitudes

The highest average score of “Willing to get nutrition information” and “Prefer to eat healthy food” was found compared to other attitude related food and nutrition. About 46% of reproductive women “prefer to eat nutrition food”. About 63% of reproductive women were willing to get information about nutrition. About 57% of reproductive age women would like to buy healthy nutritious food, but only 33% of women paid attention to nutritious food. A statistically significant difference was found among healthy food attitudes for women based on area, a residential period of the study area, family size, family income and level of education (*p* < 0.05). In this result, all attitude scores were higher for monthly income (see Table 5).

### 3.4. Food and Nutrition-Related Practices

The study showed that a high number of rural women preferred traditional Sri Lankan dietary patterns. Over half of the rural participants (58.5%) claimed to like to buy fresh food. However, most of the women in both areas (57.5%) did not care about the quality of food. Most urban women (41.5%) preferred to buy processed foods, but rural women avoided them. The majority of marginalised women usually cared about food prices (90.75%) and food taste (95.5%). Of urban women, 77.5% cared about the food-preparation method when they were buying food, which is a much higher percentage as compared with rural women. From the mean percentage of positive responses on the three attitude subscales, the study found that rural women expressed favourable attitudes towards traditional Sri Lankan foods. More than 50% of reproductive-age women like to eat traditional Sri Lankan food, while women who had a high level of education expressed more positive attitudes about nutrition in general. Two-thirds of participants (66%), most of them rural women (97%), stated that they usually eat breakfast. Just over half of participants (51.3%), majority urban (63.5% of urban women), usually skip lunch. All participants typically eat dinner in the evening. Just 45.5 % reported eating all three meals regularly. Statistically significant differences were found in “Type of diet practice”, “What do you usually eat for your main meal?” and “prefer to buy fresh food” (*p* < 0.05) (see Table 6).

### 3.5. Multiple Linear Regression to Identify Factors That Affect Food and Nutritional Knowledge

In multiple-linear-regression models, a significant difference was found in the area, age, family size, monthly income, educational level, attitudes towards nutrition and nutrition practices across the reproductive women (R^2^: 0.467; *p* < 0.01). In this sample, reproductive women acquire more nutrition knowledge as they grow older. Women who live in rural areas had more knowledge of nutrition than those living in urban areas because of their traditional nutritional knowledge. Additionally, this sample found more women over 25 years of age residing in rural areas than urban areas. Women who had the highest education level had better knowledge about nutrition. Women who paid attention to nutrition practice and attitude had more knowledge than those who did not pay attention to nutrition. There was no statistical significance between marital status, residency period of living area, nutrition-related behaviour, and perception of food quality in reproductive-age women in marginalised areas. In this sample women who have born in the residential area were 74% and 26% of women migration from the same province that they are living. Therefore, the residency period in the living area did not impact on knowledge level (see Table 7).

### 3.6. Risk-Factor Analysis

Knowledge, Attitudes, and Practices (KAP) questions were scored as appropriate and compared to BMI levels. A correlation matrix is used for risk-perception studies [81]. In this study, the correlation coefficient was computed to elucidate any possible relationship between variables; Table 8 shows the analysis results.

There was a highly significant positive correlation between nutritional knowledge, attitude score, and BMI levels. This indicates that self-awareness regarding serious nutritional problems and knowledge regarding micronutrition deficiencies, the importance of including vitamin-rich foods in the diet, maintaining a balanced diet, and an understanding of good nutrition and food preparation resulted in better nutritional levels.

A significant positive correlation was found between the eating-practice scores of women and BMI levels. Practices like eating three times a day, consuming vitamin-rich foods, frequency of eating fruits and vegetables, daily consumption of water, and food-preparation methods resulted in better nutritional levels for these women. Furthermore, there was a highly significant positive correlation between socioeconomic and environmental factors and BMI levels, but the residential area was not significantly correlated with BMI level (see Table 9).

### 3.7. Correlation Analysis between BMI Level and Women’s Knowledge about the Perception of Food Quality

In the survey, women’s knowledge about the perception of food quality, perceived risk and issue attributes, and perception of the food were examined. The food-choice score of the subjects was significantly correlated with their BMI level (Table 10). This indicated that their food choices significantly impact their BMI.

However, the attention-to-food-quality score was highly significantly correlated with their BMI level. This indicates that paying attention to the quality of food when purchasing or eating food can impact their nutrition (see Table 11).

## 4. Discussion

The aim of this study was to identify the food and nutrition-related KAP among women of reproductive age in marginalised areas in Sri Lanka. According to the basic principle of the KAP model, improving knowledge leads to changes in attitudes and behaviours that reduce the human and economic burden of diseases [59,82,83]. Also, the KAP model emphasizes the beneficial influence of nutrition on health promotion, diseases management and risk reduction [84]. According to the WHO, women play the main role in achieving a healthy nutrition policy both in the family and society as a whole. Furthermore, the health and status of women of reproductive age has a large impact on the health of their children and future generations [85]. Therefore, reproductive-age women are important guiders who can influence the nutrition of their household.

This survey showed that reproductive women had a limited grasp of food and nutrition-related knowledge. For these results, knowledge may be the main factor in starting changes in dietary behaviour [63]. If women have an insufficient nutrition background, they cannot prevent family nutrition problems. A study on nutrition knowledge of women in Bosnian on dietary iron intake shows low level of knowledge impact the occurrence of anaemia [36]. Upadhyay et al. [86], Charlton et al. [87] and Shahzad et al. [88] found a low level of nutritional knowledge impact on a nutritional problem such as iron and iodine deficiencies. Compared to other research, this study more accurately measured the degree of nutritional related knowledge among women. However, any interpretation of these results should consider that this study used broad standards of nutrition knowledge of reproductive-aged women. The outcomes show that most low average scores in food and nutrition-related knowledge were among undernutrition women. Also, this study sample shows a low level of knowledge about food and nutrition-related diseases. This may imply that there are misunderstandings for a range of nutrition-related knowledge. Therefore, a low level of nutrition knowledge is an issue that may impede women’s ability to consume a well-balanced diet, which will result in poor dietary intake.

Furthermore, results showed that only 33.5% of the participating reproductive-age women had normal weight, and most of the participants (more than 50%) had a nutritional problem such as under or overnutrition, or even obesity; many of them were undernourished. This showed that most reproductive-age women suffer from nutritional problems in the examined areas of Sri Lanka. Unhealthy food practices can directly or indirectly affect the quality of life and health through poor nutritional status [70,89,90]. For example, similar research in Sri Lanka found that insufficient nutrition-related knowledge is an underlying cause for the high prevalence of undernutrition and micronutrient deficiencies [39]. Food insecurity in low-income families is associated with a significantly higher percentage of diabetes in community samples, especially among women [1,91,92]. Food security requires nutritional adequacy [93,94]. This means that, in the broadest sense, any individual who is undernourished or has micronutrient deficiency can be seen as food insecure. These deficiencies show the importance of understanding the dynamics of both household and individual food security, and an unbalanced distribution of adequate amounts of food within the household, which may result in deficiencies [95]. On the other hand, reproductive-age women are at particular risk of poor health due to undernutrition and micronutrient deficiencies in general and during pregnancies. Reproductive-age food insecurity has also been associated with poor pregnancy outcomes, including low birth weight and gestational diabetes [96,97]. This study demonstrated a significant correlation between the level of nutritional knowledge of reproductive-age women and BMI status. Most of the reproductive-aged women’s education level scored higher than their nutritional knowledge. The study found that women with a higher education level had better knowledge about nutrition [70] and were mindful about their food and nourishment. This study indicated that, through nutritional knowledge, women can change their food behaviour. It may help eliminate nutritional problems in these areas. Furthermore, women being are a key factor of the household food and nutrition security especially in developing countries so that women’s health and knowledge should be given significant consideration which will assist in attaining Millennium Development Goals (MDGs). Many studies have supported encouraging healthy behavioural practices among women [37,98].

The study results show that many women “prefer to eat nutritious food” and are “willing to get information about nutritious food”. Also, more than 50% of women are willing to buy nutritious food. This means the there is a positive approach to running nutrition education programs in these areas. But attention to eating and preparing nutritious food scores was low-level. Poverty may affect attitude and practice. This study showed that the attitude scores of women were also significantly correlated with their BMI level in these areas. A similar research study found that there was a relationship between positive attitude and healthy eating practices [99]. More than 50% of women stated in the survey that they liked traditional food and food flavours, but most of them were not interested in nutrition at all. However, this study proves once more that food practices have an impact on the BMI status of women. Many urban women prefer to eat processed food than taste traditional fresh food. For example, one of the women (interviewee in a metropolitan slum area) stated that her husband was working in a Middle Eastern country. When she receives money from her husband, she goes to a McDonald´s restaurant with her child. Unfortunately, she had first-stage cancer. She may not understand that processed food may affect her health situation, but she does not change her food practices. This is one example of the current situation of food attitudes in Sri Lanka. Poverty is especially a problem for marginalised societies. Results in these observed areas help to better understand personal determinants of food habits and detect relevant nutritional problems. This may impact nutritional status and the health situation of families in this region. According to the current study, reproductive-age women suffer from a double burden of malnutrition that can increase the risk of cancer, diabetes, high blood pressure, and cholesterol. The reason for these problems can be viewed as a result of a low level of nutritional knowledge. After the Green Revolution, there was a replacement of vegetable-based foods in Sri Lankan food culture with animal-based foods, and increased consumption of sugar, salt, and alcohol, surpassing the recommended intake. A plant-based diet reduces the risk of developing obesity, diabetes, cardiovascular diseases, and some forms of cancer [100,101]. Unfortunately, in Sri Lanka, people do not understand or know less about traditional food culture. Hence, nutritional status is influenced by multiple and inter-related factors. For this purpose, KAP studies help to evaluate and investigate people’s KAP relating to nutrition, diet, food, and health issues [102].

Food and nutrition-related behaviours directly have an impact on their knowledge level. This study shows the perception of food quality was low scoring in the study sample. Some of the participants understood the perceived risks of unhealthy food. Still, most were unconcerned about such risks because marginal societies suffer from poverty or they have barriers to eating healthy food. This study demonstrated that, in slum areas, most women do not prepare food themselves. Many of them said that preparation of food at home is costlier than buying prepared foods because in these areas, they can easily buy cheap unhealthy food (including fats, especially of animal origin), “fast” food (that is low in fibre and vitamins), foods high in salt and tropical oils (e.g., patties, rolls and sauces). Although this food is not nutritious (adding more essence of taste, “ajino moto”) it was regarded as tasty. It was also observed that most of them had very small kitchens that were hygienically unsafe (poor sanitation, low spaces, and no cleaning). This also may be the results of low-level food and nutrition knowledge and practice.

In addition to education, having a low socioeconomic status puts the family at risk of having poor diet, which can subsequently compromise their growth and development, especially for vulnerable groups such as children and women. Family income, a frequently used indicator of socioeconomic status, has been consistently shown to influence diets. A study result shows that high income may associated with a better quality of diet [103]. A higher income could mean stronger purchasing power for better quality of foods, while a limited income restricts access to nutrient-dense foods. In this study, 4.3% of participants had a monthly income of less than Rs 5000 (1 United States dollar = 185 Sri Lankan rupees). Most of them were part of rural households. More than 50% were low-income dwellers. This indicates a positive correlation between income and BMI (correlation = 0.172, *p*-value = 0.001). However, a monthly income of more than Rs 35,000 might indicate undernutrition problems for women, too. Previous studies showed that low socioeconomic status impact on lacked knowledge of healthy nutrition [104].

By employing multiple-linear-regression models, a significant difference was found among the factors of age, family size, monthly income, educational level, attitudes towards nutrition, and food and nutrition practices across reproductive women. Another notable point is that most women aged 35–49 years or older gained nutritional knowledge from their elders, which means that traditional indigenous knowledge is important for nutritional status. Even today, older people are well-aware of their food-security risks [53]. Local natural-food knowledge and resources still exist [105]. There is a gap of acknowledgement and acceptance of traditional and local knowledge between generations. Field results revealed that most women in marginalised rural areas use their traditional knowledge and diverse food resources to improve their own nutrient status. However, this knowledge is not documented and creates a knowledge gap between older and younger generations about nutritional values. Therefore, this knowledge must be secured for the future [106]. Previous study about women’s nutritional traditional of food could also impact the nutritional status of the family [29]. Therefore, knowledge may be a powerful indicator [107] to maintain household nutritional security.

This KAP study provided a better understanding of women´s personal determinants of dietary behaviour and valuable information on planning programmes to prevent the risk of reproductive-age women in marginalised areas. These results could be used to plan culturally appropriate diet- and lifestyle-counselling programs for the management of women with nutritional problems. The findings of this study may be important for future nutrition policymakers. Research that focuses on reproductive-age women may play an important part in health promotion and the prevention of nutrition-related health problems, but it requires education. Some suggestions exist for improving the nutrition-related knowledge of reproductive-age women to improve and meet the health-education demands of households. First, training for nutrition-related teaching, especially traditional-food and nutrition-related knowledge, should be strengthened in women and be part of the national educational curriculum. If rural and urban women have space for a garden, knowledge about plants and their nutritional benefits might be helpful as well. School gardens might inspire boys and girls to learn about food [108]. Second, with regard to women who are younger and are underweight, it would be beneficial to adopt corresponding protocols for the screening and education of these groups living in urban and rural areas by a nutritionist or dietitian. Third, the media network can be used as a new way to spread traditional indigenous knowledge to women. For example, showing traditional nutritional food-preparation methods, introducing ways to prevent some health problems with traditional food (traditional Sri Lankan food has more benefits to prevent noncommunicable diseases), and different harvesting and storage methods. It would be beneficial not only for improving family nutrition, but also in promoting an overall more sustainable and health-supporting food system in Sri Lanka.

There are several limitations to this study. The study uses the KAP model which is a first-generation approach in health behavior research. These days third-generation theory-based approaches or fourth-generation multi-theory-based approaches are being used in health behavior research. This model comprises prefined questions that capture information on critical knowledge, attitude and practice related to the most common nutritional issues [103]. This model emphasizes the beneficial influence of nutrition on health promotion, disease management and risk reduction. Data obtained through a cross-sectional survey did not permit to determine the causality. Both areas were big, and the sample size was relatively small which may have impacted the accuracy and reliability. Hence, the investigators received training for the control of data. This research investigated two different marginalised communities. In this case, a marginalised community means vulnerable people in Sri Lankan society. Reproductive-age women are a good indicator of the overall well-being of society. Furthermore, because the study was examined in two different marginalised areas of reproductive-age women, the KAP results cannot be generalised to other settings. Therefore, the study sample was confined to reproductive-age women. They are vulnerable because they have had nutritional problems and health and social issues. Furthermore, the evaluation of this study among reproductive-age women was self-reported. This study investigated the association between nutritional knowledge and demographic factors with nutrition status that was represented by BMI. This research did not investigate the nutritional status.

## 5. Conclusions

This study on reproductive-age women in Sri Lanka living in urban and rural marginalised areas is the first to use the KAP model. In this study, we examined the influence of dietary knowledge, attitudes, and practices (KAP) among reproductive-age women in marginalised areas in Sri Lanka. The research results clearly showed that the reproductive age women have a low level of nutritional knowledge in marginalized areas in Sri Lanka. Most women have a positive attitude about receiving nutritional knowledge but have low-level practice about a healthy diet. Furthermore, knowledge, practices, and attitudes of women largely affect their BMI status, as well as household food security. On the basis of multiple-linear-regression analysis, residency period of living area, nutrition-related behaviour, and perception of food quality in reproductive-age women in marginalised areas did not show a significant relationship with any of the tested risk-perception domains in the model. According to our study, in these areas, most of the reproductive age women have nutritional problems (under-or overnutrition, or obesity) and this may affect future nutritional insecurity, as well as the unborn children and the health condition of these women. The establishment of dietary guidelines is not sufficient to ensure that women are equipped with the knowledge necessary to optimize their diets for the health of reproductive-age women and their unborn babies. Healthcare or nutrition information providers have an important role in promoting knowledge of healthy eating. On the basis of the results of this research, there is a need to enhance nutritional education in reproductive-age women. The finding will help policymakers or responsible authorities to plan appropriate nutrition care programs for vulnerable people living in marginalized areas and these results could be empowering sustainable food and nutrition system in Sri Lanka and they can provide valuable inputs for effective programme and project. Further studies should be conducted to investigate the strategic nutrient intake and micronutrient adequacy of reproductive-age women, and their dietary diversity for nutrition and health in marginalized societies in Sri Lanka.

## Figures and Tables

**Table 1:** The study questions of food and nutrition-related knowledge and attitude.

Dimension	Total Number of Items	Description	References
Dietary fiber-related knowledge	7	Which foods that contained high fibre? /Eating adequate amounts of dietary fibre can prevent and treat diseases? Which diseases can prevent and treat dietary in fibre?/Fast-food consumption, in general, contributes to our dietary fibre intake?/Regular consumption of dietary fibre may reduce blood cholesterol levels?/Dietary fibre Can help maintain our body-weight/Dietary fibres have calorie	Macías et al. [52] Deniz et al. [61] Guiné et al. [62] Bookari et al. [63]
Micronutrients- related knowledge	9	Micronutrient deficiency-related health problem/knowledge about micronutrients food: Fe, vitamin A, vitamin C, folic acid, B vitamins, vitamin E and vitamin B12/Have you ever heard or read that vitamin folic acid can help and prevent birth deceases	Macías et al. [52] Bookari et al. [63] Augustine et al. [64]
Iron-related knowledge	7	What are the health risks of a lack of iron in the diet?/What causes anaemia?/How can anaemia be prevented?/Can you list examples of foods rich in iron?/When taken during meals, certain foods help the body absorb and use iron?/What are those foods some beverages decrease iron absorption when taken with meals, Which ones?/Have you heard about iron-deficiency anaemia? If Yes: Can you tell me how you can recognize someone who has anaemia?	Macías et al. [52]
Fat-related knowledge	5	Which food in high fat? /Do you know the risk of consuming high-fat food? What is the risk of consuming a high-fat food? Which fat good for the body? /How much do you daily consuming oil and which type of oil are you using?	Macías et al. [52] Stafleu et al. [65]
Food and nutrition diseases	6	Knowledge of high blood pressure, cholesterol/Knowledge about micronutrition deficiencies is low nutrition food consumption salty snack is harmful health/Is fast food contains low nutrition or not?/Unhealthy eating patterns are the risk of future health/Do you know eating nutritious food can live a healthy life	Macías et al. [52]
Vitamins and mineral-related knowledge	7	Why vitamin and mineral are essential?/What are the most important vitamin and minerals for your body?/Which vitamin and minerals are good for bone development?/Which vitamin and minerals are good for energy production and immune function?/Why is protein important for humans? Which vitamin and minerals are good for healthy eyes and general growth and development? /What are daily essential vitamins for your body.	Macías et al. [52]
Healthy food-related knowledge	9	What is a balanced diet? /Why is a balanced diet important? Do you know the food pyramid? /What foods are healthy? What food should you eat every day?/Which foods are the healthiest?/Which foods should not eat or unhealthy?/Which cooking method is protecting nutrition in vegetables/How well do you think it is to have different types of foods at meals?	Macías et al. [52]
Healthy food attitude	8	The food I eat must keep me (healthy/nutritious/contains vitamins and minerals) / I always follow a healthy and balanced diet/I eat what I like and I do not worry about the healthiness of food/The healthiness of food has little impact on my food choices/Ranking of food choice motives (Taste, Convenience, Health & Nutrition, Price)/What I eat and what I don’t care about is the healthiness of food_?	Macías et al. [52] Naughton et al. [66]
Attention to preparing nutritious food attitude	**6**	Are you eating nutritious food every day?/When you are preparing food you are attention balanced diet?/Which type of food do you prefer to eat?/Are you preparing the main meal yourself and if yes, are you attention nutrition guidelines? How confident do you feel about preparing nutritious food for your family?	Macías et al. [52] Alvarenga et al. [67]
Willingness to buy nutritional food	2	What type of food are you willing to buy?/When you are buying food in the markets your choices are (Taste, Convenience, Health & Nutrition, Price)	Macías et al. [52] Huang & Chung [68] Tsakiridou et al. [69]
Satisfaction about eating practices	2	How confident do you feel about preparing nutritious food for your family? Are you satisfied with daily meal patterns and knowledge?	Macías et al. [52] Liu et al. [70]
Willing to get nutrition information	2	Are you willing to learn more knowledge about healthy food and nutrition? Would you like to join nutrition education programs in your region?	Macías et al. [52] James [71] Prelip et al. [72]

**Table 2:** Demographic characteristics of reproductive-age women (*N* = 400).

Variables	Category	*N*	%
Type of residence	Rural	200	50.0
	Urban	200	50.0
Age	15–25	141	35.3
	26–35	141	35.3
	36–49	118	29.5
Marital status	Married	328	82.0
	Single	30	7.5
	Separated	18	4.5
	Widowed	24	6.0
Family members *	Small family	260	65.0
	Big family	140	34.5
Level of education	No education	37	9.3
	Primary	234	58.5
	Secondary	125	31.3
	Higher	4	1.0
Main occupation	Unemployed	241	60.5
	Agriculture	68	17.0
	Hard job	79	19.8
	Government job	11	2.8
Monthly family income ^1^	Less than 5000	17	4.3
	5000–15,000	138	34.5
	15,001–25,000	100	25.0
	25,001–35,000	71	17.8
	35,001–50,000	57	14.3
	More than 50,000	17	4.3
BMI	Underweight	174	43.5
	Normal weight	134	33.5
	Overweight	77	19.3
	Obese	15	3.8
Residence in the study area	From birth	296	74.0
	Immigrated from somewhere	104	26.0

* According to the “small family is golden’’ family-planning concept in Sri Lanka. In this case, “small family” means a father, a mother, and two children (minimum four family members), “Big family” means a father, a mother and more children (more than four family members) [80]. ^1^ Sri Lankan rupees per month.

**Table 3:** Each dimension of the average score indifference (knowledge) variables (Mean, SD) (*N* = 400).

Variables	1	2	3	4	5	6	7
Age							
15–25	0.72 ± 0.449	0.33 ± 0.473	0.02 ± 0.145	0.94 ± 0.830 *	0.72 ± 0.449	0.91 ± 0.280 *	0.98 ± 0.145
26–35	0.77 ± 0.420	0.29 ± 0.473	0.06 ± 0.245	0.73 ± 0.933 *	0.77 ± 0.420	0.67 ± 0.471 *	0.93 ± 0.258
36–49	0.81 ± 0.398	0.21 ± 0.410	0.04 ± 0.202	0.44 ± 0.801 *	0.81 ± 0.398	0.83 ± 0.377 *	0.97 ± 0.182
Area							
Rural	0.70 ± 0.462	0.40 ± 0.490 *	0.06 ± 0.229	1.02 ± 0.905 *	0.70 ± 0.462 *	0.65 ± 0.480 *	0.95 ± 0.218
Urban	0.84 ± 0.372	0.17 ± 0.377 *	0.03 ± 0.171	0.42 ± 0.746 *	0.84 ± 0.372 *	0.97 ± 0.184 *	0.97 ± 0.184
Household size							
Small family	0.64 ± 0.480 *	0.40 ± 0.491 *	0.07 ± 0.248 *	0.96 ± 0.908 *	0.64 ± 0.480 *	0.72 ± 0.452 *	0.93±0.248 *
Big family	0.99 ± 0.085 *	0.06 ± 0.246 *	0.00 ± 0.000 *	0.26 ± 0.607 *	0.99 ± 0.085 *	0.97 ± 0.167 *	1.00±0.000 *
Marital status							
Married	0.72 ± 0.448	0.32 ± 0.468 *	0.05 ± 0.209	0.80 ± 0.894 *	0.72 ± 0.448 *	0.77 ± 0.421	0.95 ± 0.209
Single	0.93 ± 0.254	0.20 ± 0.407 *	0.07 ± 0.254	0.73 ± 0.907 *	0.93 ± 0.254 *	0.90 ± 0.305	0.93 ± 0.254
Separated	0.94 ± 0.236	0.06 ± 0.236 *	0.00 ± 0.000	0.00 ± 0.000 *	0.94 ± 0.236 *	1.00 ± 0.000	1.00 ± 0.000
Widowed	1.00 ± 0.000	0.00 ± 0.000 *	0.00 ± 0.000	0.17 ± 0.482 *	1.00 ± 0.000 *	0.09 ± 0.302	1.00 ± 0.000
Main Occupation							
Unemployed	0.69 ± 0.465 *	0.37 ± 0.468 *	0.02 ± 0.143 *	0.88 ± 0.908 *	0.69 ± 0.465 *	0.74 ± 0.437	0.98±0.143 *
Agriculture	0.90 ± 0.465 *	0.18 ± 0.384 *	0.04 ± 0.207 *	0.69 ± 0.815 *	0.90 ± 0.306 *	0.91 ± 0.286	0.97±0.170 *
Hard job	1.00 ± 0.000 *	0.00 ± 0.000 *	0.00 ± 0.000 *	0.05 ± 0.273 *	1.00 ± 0.000 *	1.00 ± 0.000	1.00±0.000 *
Government Job	0.82 ± 0.405 *	1.00 ± 0.000 *	0.82 ± 0.405 *	2.00 ± 0.000 *	0.00 ± 0.000 *	0.09 ± 0.302	0.09±0.302 *
Monthly income							
>5000	1.00 ± 0.000 *	0.00 ± 0.000 *	0.00 ± 0.000 *	0.41 ± 0.795 *	1.00 ± 0.000 *	1.00 ± 0.000 *	1.00±0.000 *
5000–15,000	0.96 ± 0.205 *	0.05 ± 0.220 *	0.00 ± 0.000 *	0.20 ± 0.511 *	0.96 ± 0.205 *	0.96 ± 0.180 *	1.00 ± 0.000 *
15,001–25,000	0.83 ± 0.378 *	0.21 ± 0.409 *	0.00 ± 0.000 *	0.64 ± 0.785 *	0.83 ± 0.378 *	0.94 ± 0.239 *	1.00 ± 0.000 *
25,001–35,000	± 0.495 *	0.54 ± 0.503 *	0.04 ± 0.203 *	1.04 ± 0.948 *	0.59 ± 0.495 *	0.75 ± 0.438 *	0.97 ± 0.167 *
35,001–50,000	0.56 ± 0.501 *	1.00 ± 0.000 *	0.11 ± 0.310 *	1.42 ± 0.801 *	0.56 ± 0.501 *	0.44 ± 0.501 *	0.89 ± 0.310 *
Education level							
Non-education	1.00± 0.000 *	0.00 ± 0.000 *	0.00 ± 0.000	0.19 ± 0.397 *	1.00 ± 0.000 *	1.00 ± 0.000 *	1.00 ± 0.000 *
Primary	0.76 ± 0.430 *	0.27 ± 0.447 *	0.03 ± 0.158	0.67 ± 0.869 *	0.76 ± 0.430 *	0.83 ± 0.373 *	0.97 ± 0.158 *
Secondary	0.71 ± 0.455 *	0.38 ± 0.488 *	0.09 ± 0.284	0.96 ± 0.928 *	0.71 ± 0.455 *	0.69 ± 0.465 *	0.91 ± 0.284 *
Higher	0.75 ± 0.500 *	0.25 ± 0.500 *	0.00 ± 0.000	1.00 ± 0.816 *	0.75 ± 0.500 *	1.00 ± 0.000 *	1.00 ± 0.000 *
BMI							
Underweight	0.87 ± 0.333 *	0.18 ± 0.389 *	0.00 ± 0.000 *	0.54 ± 0.742 *	0.87 ± 0.333	0.99 ± 0.076 *	1.00 ± 0.000 *
Normal weight	0.66 ± 0.477 *	0.38 ± 0.487 *	0.09 ± 0.287 *	0.93 ± 0.943 *	0.66 ± 0.477	0.65 ± 0.479 *	0.91 ± 0.287 *
Overweight	0.71 ± 0.455 *	0.34 ± 0.476 *	0.05 ± 0.223 *	0.77 ± 0.972 *	0.71 ± 0.455	0.62 ± 0.488 *	0.95 ± 0.283 *
Obese	0.73 ± 0.458 *	0.26 ± 0.458 *	0.07 ± 0.258 *	0.60 ± 0.910 *	0.73 ± 0.425	0.93 ± 0.258 *	0.93 ± 0.258 *
Average mean score	0.77 ± 0.425	0.28 ± 0.451	0.04 ± 0.202	0.72 ± 0.880	0.77 ± 0.425	0.81 ± 0.397	0.96 ± 0.202

(1) Dimensions: 1- Iron-related knowledge, 2- Micronutrients related knowledge, 3- Nutrient and food-related diseases, 4- Healthy food-related knowledge, 5- Fat- related knowledge, 6- Vitamins and mineral-related knowledge, 7- Dietary fibre related knowledge (2) Abbreviation: SD (Standard Deviation) (3) * statistical significance (*p* < 0.05) (4) ANOVA, *t*-test, Mann Whitney U test was used to compare differences.

**Table 4:** Knowledge level of reproductive-age women in urban and rural areas in Sri Lanka regarding nutrition (*N* = 400).

Knowledge Level *	Rural Areas (*N* = 200; %)	Urban Areas (*N* = 200; %)	Total (*N* = 400; %)
Knowledge about vitamins and minerals			
Yes	79 (39.5)	34 (17)	113 (28.2)
Do not know	121 (60)	166 (83)	287 (71.8)
Knowledge about fat and diet			
Yes	61 (30.5)	33 (16.5)	94 (23.5)
Do not know	139 (69.5)	167 (83.5)	306 (76.5)
Knowledge about iron-related food			
Yes	61 (30.5)	33 (16.5)	94 (23.5)
Do not know	139 (69.5)	167 (83.5)	306 (76.5)
Knowledge about micronutrients			
Yes	31 (15.5)	31 (15.5)	62 (15.5)
Do not know	135 (67.5)	161 (80.5)	42 (10.5)
Not well	34 (17)	8 (4)	296 (74)
Knowledge about food and nutrients			
No	80 (40)	147 (73.5)	227 (56.8)
Not well	37 (18.5)	22 (11)	59 (14.8)
Yes	83 (41.5)	31 (15.5)	114 (28.5)
Knowledge about dietary fibre			
No	189 (74)	194 (97)	383 (95.8)
Yes	11 (5.5)	6 (3)	17 (4.2)
Knowledge about water and food consumption			
Yes	122 (61)	72 (36)	194 (48.5)
No	78 (39)	128 (64)	206 (51.5)
Knowledge about food and nutrition-related diseases			
Yes	71 (35.5)	7 (3.5)	78 (19.5)
No	129 (64.5)	193 (96.5)	322 (80.5)

* According to Preliminary analysis.

**Table 5:** The average score of food and nutrition-related attitude (mean, SD).

Variables	1	2	3	4	5
Area	0.37 ± 0.484 *	0.25 ± 0.436 *	0.13 ± 0.340 *	0.27 ± 0.445 *	0.50 ± 0.501 *
Residental period	0.34 ± 0.475 *	0.50 ± 0.502 *	0.64 ± 0.484 *	0.43 ± 0.497 *	0.26 ± 0.439 *
Age	0.81 ± 0.821 *	0.85 ± 0.766	1.02 ± 0.695	0.93 ± 0.789	0.44± 0.804 *
Family size	1.21 ± 0.406 *	1.05 ± 0.210 *	1.10 ± 0.300 *	1.10 ± 0.303 *	1.35± 0.478 *
Marrital status	0.44 ± 0.898	0.05 ± 0.258 *	0.02 ± 0.128 *	0.01 ± 0.120 *	0.35 ± 0.823
Main occupation	0.73 ± 0.922	0.31 ± 0.822 *	0.49 ± 1.045 *	0.31 ± 0.733 *	0.65 ± 0.887
Family income	2.51 ± 1.316 *	3.19 ± 1.229 *	3.51 ± 1.233 *	3.12 ± 1.209 *	2.16 ± 1.274 *
Level of education	1.45 ± 0.567 *	1.33 ± 0.588 *	1.48 ± 0.536 *	1.36 ± 0.626	1.24 ± 0.623 *
BMI level	1.78 ± 0.737	1.98 ± 0.718	2.23 ± 0.643	2.10 ± 0.840 *	1.83 ± 0.867 *
Average score of attitude	2.35 ± 1.039	1.34 ± 0.470	1.99 ± 0.655	1.66 ± 0.476	2.54 ± 1.316
Perentage of women	46	33	57	35	63

1. Prefer to eat a healthy food attitude, 2. Attention to prepare nutritious food, 3. Willing to buy nutritious food, 4. Satisfaction about eating practice 5. Willing to get nutrition information, Abbreviation: SD (Standard Deviation) * statistical significant (*p* < 0.05).

**Table 6:** Reproductive-age women’s food practices in rural and urban areas of Sri Lanka (*N* = 400).

Variables	Rural	Urban	Total	Mean ± SD
Type of diet practice				
• Traditional Sri Lankan diet	187	23	210	0.11 ± 0.313 *
• Fried food	13	162	175	0.93 ± 0.263 *
• Wheat products	0	15	15	1.00 ± 0.000 *
What do you usually eat for your main meal?				
• Different choices	0	3	3	1.00 ± 0.000 *
• Mostly rice and curry	33	19	52	0.37 ± 0.486 *
• Depends on daily income	73	174	247	0.66 ± 0.476 *
• Only rice and curry	94	4	98	0.04 ± 0.196 *
Do you use traditional food flavours?				
• Yes	167	63	230	0.25 ± 0.447 *
• No	33	137	170	0.81± 0.393 *
What type of food do you prefer to buy?				
• Fresh food	83	5	88	0.06 ± 0.233 *
• Anything	117	112	229	0.49 ± 0.501
• Processed food	0	83	83	1.00 ± 0.000
Daily eating patterns				
• Usually, eat breakfast	194	70	264	0.27 ± 0.442 *
• Usually, eat lunch	122	73	195	0.37 ± 0.485 *
• Usually, eat dinner	200	200	400	0.50 ± 0.501
• Usually, eat three times	116	66	182	0.36 ± 0.482 *
Eating habits				
• Eating in front of the TV	89	40	129	0.37 ± 0.242 *
• Eating with family members	10	11	21	0.07 ± 0.285
• Eating on the way	25	66	91	0.39 ± 0.501
• Not a special	66	83	149	0.50 ± 0.480 *

Abbreviation: SD = standard deviation; * statistically significant (*p* < 0.05).

**Table 7:** Multiple linear regressions to identify factors that affect food and nutritional knowledge of reproductive-age women in rural and urban areas of Sri Lanka (*N* = 400).

Variables	β	SE	VIF	*t*	*p*-Value
Age	0.256	0.050	1.388	5.091	0.000 *
Area	0.062	0.095	2.258	1.125	0.049 **
Marital status	−0.127	0.012	3.574	−2.391	0.115
Family size	0.542	0.089	1.809	6.092	0.000 *
Main occupation	0.352	0.049	1.970	−2.335	0.029 **
Monthly income	0.406	0.040	1.417	2.007	0.001 *
BMI	0.118	0.011	1.534	2.391	0.027 **
Residency period in living area	−0.002	0.022	2.433	−0.242	0.966
Educational level	0.469	0.141	1.505	3.330	0.001 *
Attitudes towards nutrition	0.049	0.086	2.510	−0.573	0.000 *
Food and nutrition practice	0.208	0.128	1.216	−1.627	0.020 **
Nutrition-related behaviour	−0.689	0.144	2.596	−4.773	0.273
Perception of food quality	−0.105	0.303	2.916	−0.346	0.495

Abbreviation: β-Beta weights, SE-Std. Error, ** Correlation significant at 0.01 level (two-tailed); * correlation significant at 0.05 level (two-tailed), VIF: variance Inflation Factor.

**Table 8:** Correlation analysis between the knowledge level of reproductive-age women in Sri Lanka and BMI levels.

Variables	Mean	SD	Correlations	*p*-Value
BMI level ^1^	1.83	0.867		
Nutrition-knowledge score	0.04	0.190	0.129 **	0.005
Attitude score	1.55	0.758	−0.283 **	0.000
Practice score	0.33	0.500	0.117*	0.020

** Correlation significant at 0.01 level (two-tailed); * correlation significant at 0:05 level (two-tailed). ^1^ BMI [underweight (undernutrition), normal weight, overweight (overnutrition), obese (obesity)].

**Table 9:** Correlation analysis between socioeconomic and environmental factors and BMI.

Variables	Mean	SD	Correlations	*p*-Value
BMI	1.83	0.867		
Monthly income	2.16	1.274	0.172 **	0.001
Age	0.94	0.804	0.278 **	0.000
Main occupation	0.65	0.889	−0.109 *	0.000
Family size	1.35	0.479	0.178 **	0.000
Residential area	0.50	0.501	−0.026	0.604

** Correlation significant at 0.01 level (two-tailed); * correlation significant at 0.05 level (two-tailed).

**Table 10:** Correlation analysis between BMI levels and reproductive-age women’s perception of food choices.

Variables	Mean	SD	Correlations	*p*-Value
Perception of food choices	1.10	0.324	0.158 *	0.002
BMI levels	1.83	0.867		

* Correlation significant at 0.01 level (two-tailed).

**Table 11:** Correlation analysis between BMI levels and reproductive-age women’s perception of food quality.

Variables	Mean	SD	Correlations	*p*-Value
Perception of food quality	1.55	0.758	−0.283 *	0.000
BMI levels	1.83	0.867		

* Correlation significant at 0.01 level (two-tailed).
